# Mechanisms of Testicular Disruption from Exposure to Bisphenol A and Phtalates

**DOI:** 10.3390/jcm9020471

**Published:** 2020-02-08

**Authors:** Francesco Pallotti, Marianna Pelloni, Daniele Gianfrilli, Andrea Lenzi, Francesco Lombardo, Donatella Paoli

**Affiliations:** 1 Laboratory of Seminology—Sperm Bank “Loredana Gandini”, Department of Experimental Medicine, “Sapienza” University of Rome, Viale del Policlinico 155, 00161 Roma, Italy; francesco.pallotti@uniroma1.it (F.P.); marianna.pelloni@uniroma1.it (M.P.); andrea.lenzi@uniroma1.it (A.L.); francesco.lombardo@uniroma1.it (F.L.); 2 Department of Experimental Medicine, “Sapienza” University of Rome, Viale del Policlinico 155, 00161 Roma, Italy; daniele.gianfrilli@uniroma1.it

**Keywords:** bisphenol A, phthalate esters, spermatogenesis, testosterone, male infertility, Leydig cells, Sertoli cells, spermatozoa

## Abstract

Great attention has been paid in recent years to the harmful effects of various chemicals that interfere with our natural hormone balance, collectively known as endocrine-disrupting chemicals (EDCs) or endocrine disruptors. The effects on the reproductive system of bisphenol A (BPA) and phthalates have received particular attention: while they have a short half-life, they are so widespread that human exposure can be considered as continuous. Evidence is often limited to the animal model, disregarding the likelihood of human exposure to a mixture of contaminants. Data from animal models show that maternal exposure probably has harmful effects on the male fetus, with an increased risk of urogenital developmental abnormalities. After birth, exposure is associated with changes in the hypothalamic-pituitary-testicular axis, hindering the development and function of the male genital pathways through the mediation of inflammatory mechanisms and oxidative stress. The epidemiological and clinical evidence, while generally confirming the association between reproductive abnormalities and some phthalate esters and BPA, is more contradictory, with wildly different findings. The aim of this review is therefore to provide an update of the potential mechanisms of the damage caused by BPA and phthalates to reproductive function and a review of the clinical evidence currently available in the literature.

## 1. Introduction—The Endocrine Disruptor “Dilemma”

The Endocrine Society currently defines an endocrine-disrupting chemical (EDC) as “an exogenous chemical, or mixture of chemicals, that interferes with any aspect of hormone action” [1]. A key concept in the study of EDCs is that their mechanism of action cannot be easily demonstrated in vivo, as their effects can appear after prolonged/continuous exposure to a low dose and, even worse, these effects are often the result of the simultaneous interaction of several substances (mixture effect) and the hormone balance of the individual concerned [2]. The biological effects of EDCs may vary considerably. Many act as estrogenic/antiandrogenic compounds, involving different molecular pathways and networks after binding or mimicking the actions of either the estrogen receptor (ER) or the androgen receptor (AR), ultimately influencing cell apoptosis, proliferation, differentiation, carcinogenesis and inflammation [3]. Contamination is almost inevitable as water and foods, everyday objects (cosmetics, plastic tools and toys, etc.) and even domestic and occupational furnishings and tools (insulation, flame retardants, etc.) are a potential source of contact. 

In addition, adipose tissue can accumulate EDCs, as most of them have some degree of lipophilicity [4]. Lipolysis causes a constant release of these lipophilic EDC mixtures from adipocytes, and under certain conditions, such as obesity with dysfunctional adipocytes or weight loss, this release rate may further increase, with repercussions for endocrine/metabolic diseases or even cancer [5,6]. 

Spermatogenesis is a complex differentiation process culminating in the production of spermatozoa [7] which requires the functional coordination of numerous endocrine and paracrine factors. For this reason, EDCs have been repeatedly linked to its disruption [8,9]. Apart from their estrogenic activity, EDCs also seem to affect spermatogenesis through impairment of steroidogenesis (downregulation of CYP11A and CYP17A expression) and through the induction of oxidative stress (DNA damage and alteration of Sertoli cell tight junctions) [10]. Bisphenol A (BPA) and phthalate esters are among the most widely investigated EDCs in relation to reproductive dysfunctions and impaired spermatogenesis, but evidence is often limited to animal models and the results are controversial, often disregarding the likelihood of human exposure to a “cocktail” of contaminants [11]. In fact, with the notable exception of severe contamination after industrial accidents [12], human EDC-related diseases are more likely to be the result of long-term exposure to low (even nanomolar or picomolar) concentrations of EDC mixtures. The late onset of the resulting clinical disorders (often many years later, or even trans-generational), the complex interactions and the nonlinear dose-response all hinder the recognition of a causal relation and the establishment of tolerable threshold levels or protection [11,13,14,15]. While in vitro evidence focuses on one or a handful of EDCs with a similar activity profile, in everyday life a varied combination of EDCs can act simultaneously and may also involve modulated or antagonistic interactions, making it impossible to predict the net biological effect of the mixture [15,16].

There is a great body of published evidence on various molecular aspects of the action of endocrine disruptors and their associations with urogenital diseases. However, these studies often focus on a single or single class of EDC, meaning that the real risk to human health may be underestimated [17]. Numerous studies have concentrated on the in vivo or in vitro effects of phenols and phthalate esters, ignoring their probable interactions with other chemicals that interfere with the same molecular pathways. Given the urogenital changes induced by EDCs, future studies should not be limited to a single class of EDCs but should include at least a selection of potentially antiandrogenic chemicals (phthalate esters, AR antagonists or steroidogenesis inhibitors such as BPA, dioxin pollutants such as PCB 169, and medications such as finasteride, simvastatin or various painkillers) [17].

In light of all this, the aim of this narrative review is to provide an update of the possible ways that EDCs with a high exposure risk (phthalates and bisphenols) can disrupt reproductive function, especially testicular function, and what repercussions these might have on fertility in adulthood.

## 2. Bisphenol A and Phthalate Esters

The risk of exposure to various chemicals commonly used in everyday products, such as bisphenols (especially BPA) and phthalates, has been a cause of concern for many years. This has resulted in laws and regulations that limit their use in particularly high-risk products, such as those intended to come into contact with children (baby products, bottles, teething rings, etc.) [18]. However, these restrictions seem to be inadequate and there is still a very high risk of coming into contact with these chemicals. BPA and the most common phthalate esters are well characterized in the literature in terms both of their chemical structure and their main harmful effects on the reproductive endocrine system.

Bisphenol A (BPA) is a synthetic compound with two phenol rings connected by a methyl bridge [19]. Its use in a wide range of everyday products (food packaging, toys, plastics, etc.) is so extensive that human exposure can be considered as almost continuous [1]. This ubiquity, together with the fact that it is not chemically bound to the materials in which it is used, means that human exposure is commonplace. 

BPA can affect the male reproductive system at different levels. The available evidence suggests that exposure is associated with the decreased proliferation, increased ROS-mediated damage and increased apoptosis of male gametes through the inhibition of anti-apoptotic pathways such as Bcl-2 and activation of pro-apoptotic signalling (MAPK, Fas/FasL, Caspase 3 and 9, Bax, etc.) [19]. BPA can also disrupt the hormonal milieu of spermatogenesis. BPA itself is an AR antagonist: it acts by interfering with AR binding, reducing AR translocation and enhancing AR transcriptional corepressors [20]. Furthermore, despite its weak affinity for ERα and ERβ, low doses of BPA can trigger estrogen-like responses through different pathways [21]. BPA has been reported to interfere with testicular steroidogenesis [22,23]. 

Phthalates are widespread in industrial processes and commercial goods. Their esters can migrate out of both hard and flexible plastics (polyvinyl chloride plastics, food packaging, processing materials, toys, etc.), personal care products, solvents and even medicine film coatings [24]. In vitro studies show that phthalate esters may exert both estrogenic and antiandrogenic activity (through interactions with AR, ERα and ERβ), but their net biological effect on the hypothalamus–pituitary–gonadal (HPG) axis may vary (due to additive or synergistic effects), depending on the specific compound or mixture [25]. The final effect may also arise from disruption of ERα/ERβ tissue-specific differential expression and function [26,27]. 

As the balance of sex hormones (testosterone and estradiol) and gonadotropins is critical for the initiation and progression of spermatogenesis, disruption of the HPG axis by exposure to phthalate esters and BPA may be detrimental to sperm quality and function, as reported in both in vitro and animal studies [19,28,29].

### 2.1. Pre-Natal Exposure: Can Testicular Function Already be Compromised in Utero?

Despite the difficulties involved in evaluating EDCs, some data are available on the most pervasive. Phthalate esters and phenol species are known to negatively affect androgenic signaling, and despite their different chemical structures, they could act together to influence androgenic pathways [30], with different effects depending on the age of the individual with whom they come into contact. 

Maternal exposure during fetal life offers the greatest risk to the developing organism: exposure in this phase is associated with disruption of testosterone production and reproductive tract development, as demonstrated by the in vivo and in vitro detection of multiple antiandrogenic EDCs in fetal life [17,31,32]. This is the phase in which sex is determined, with differentiation of the reproductive tissues, a fundamental step for the future development and maintenance of reproductive function. In the male fetus, these processes are dependent on the initiation and maintenance of androgen activity. This may be disrupted by EDCs at various levels, including AR antagonization and interference with the synthesis of steroid hormones [33,34]. Disruption of these processes may thus lead to a risk of damage to the reproductive tissues while the fetus is still in the womb, leading to malformations (hypospadias, cryptorchidism, testicular hypertrophy, reduced anogenital distance), impairment of future fertility (altered spermatogenesis, infertility) and/or even the appearance of testicular tumors [32]. 

In a mouse model, it was demonstrated that the administration of different doses (0, 250, 500, 750 and 1000 mg/kg/day) of di-n-butyl phthalate (DBP) during pregnancy caused a dose-dependent testosterone reduction and an increase in the incidence of hypospadias and other congenital urogenital malformations (cryptorchidism, reduced anogenital distance) in the offspring of the 500 and 750 mg/kg/day groups compared with the controls and groups treated with the lowest dose, while a dose of 1000 mg/kg/day was fatal to the fetuses [35]. Further investigation in the offspring of pregnant rats treated with a dose of 750 mg/kg/day confirmed an increased incidence of congenital malformations (around 50% of hypospadias and other malformations, including of organs not involved in reproduction) and a downregulation of the gene expression of some signaling molecules involved in the development of the reproductive system, beginning with the genital tubercle (sonic hedgehog molecules, bone morphogenic proteins, fibroblast growth factors, transforming growth factor β1 and transforming growth factor receptor III) [36].

In 2007, Howdeshell et al. found that the exposure of pregnant Sprague Dawley rats to DBP and di–2–ethylhexyl phthalate (DEHP) (500 mg/kg each) caused different cumulative effects, from testosterone reduction to reduced gene expression of the enzymes of steroidogenesis (*cyp11a*). The authors also affirmed that the combination of the two EDCs, in comparison with administration of the individual compounds, was associated with a higher incidence of reproductive system malformations, including malformations of the epididymis, deferens and seminal vesicles and changes to the gubernaculum [33].

A more recent study by Barakat et al. (2017) found a significant age- and dose-dependent reduction in the fertility of the offspring of pregnant rats administered DHEP compared to the controls. The decline in fertility was earlier in rats exposed to high doses of DHEP (750 mg/Kg/day) and was associated with greater changes in the sex hormones and greater histological degeneration of the seminiferous tubules and the epididymis. Signs of germ cell apoptosis were also more evident in the treated group than in the controls, suggesting an association between prenatal exposure to DHEP and premature reproductive senescence induced by epigenetic changes in the pool of adult Leydig cell precursors [37]. More recently, the same authors reported that a mixture of phthalate esters similar to that found in the urine of pregnant American women caused, in the offspring of pregnant CD-1 mice, a reduction in testosterone and in the expression of mRNA of testicular steroidogenic genes (*StAR, Cyp11,* and *Cyp17*), and impaired spermatogenesis [38]. 

Further evidence suggests that exposure to these chemicals could interfere with testicular descent, inducing cryptorchidism. Testicular descent in the fetus is traditionally divided into two phases: abdominal, between gestation weeks 10 and 23, and inguinoscrotal, from week 28 to birth. The main regulatory hormones are INSL3 and testosterone. INSL3 acts predominantly in the abdominal phase, while testosterone, adjuvated by INSL3, regulates the inguinoscrotal phase, with regression of the gubernaculum testis that “guides” the testicle towards the scrotum [39]. However, the scientific evidence for EDC-mediated disruption of this system is still based above all on in vitro experiments.

Wilson et al. (2004) found that the administration of some phthalate esters (DHEP, DBP and BBP) to pregnant rats on gestation day 14 was associated with reduced testosterone production, gubernacular agenesis and reduced INSL3 miRNA expression in the fetal male rat testis (Wilson et al. 2004) [40]. The deleterious effects of DHEP on fetal Leydig steroidogenesis were confirmed in pregnant Long-Evans female rats by Lin and colleagues, who also reported that fetal exposure to DEHP has effects on fetal Leydig cell number, clustering and distribution, suggesting that abnormal expression of IGF1, KITL and LIF genes may contribute to the reproductive toxicity of phthalates [41]. Subsequently, Pathirana et al. (2011) showed that in vitro exposure to monobutyl phthalate (MBP) and DHEP inhibited hCG-induced testosterone secretion as well as INSL3 secretion from interstitial cell cultures of dog testes [42]. Furthermore, male fetal rat exposure to diisononyl phthalate (DiNP) and dicyclohexyl phthalate (DCHP) appeared to cause inhibition of gene expression and reduced protein levels of INSL3 and 3β-hydroxysteroid dehydrogenase [43], and exposure to relatively high levels of DCHP reduced the expression levels of key steroidogenesis genes [44]. 

Evidence of effects on the fetal testicular cells mainly comes from in vitro studies. Ahbab et al. (2015) found that in utero exposure to some phthalate esters (di-n-hexyl phthalate and DCHP) was associated with histological changes in the fetal testis, which presented atrophy, irregular seminiferous tubules, a reduced number or absence of germ cells and changes to the tubular walls, with the presence of multinucleated giant cells and irregular peritubular myoid cell distribution. The authors also found Leydig cell clusters of larger dimensions than those in unexposed controls [45]. Li et al. subsequently reported similar findings in the testes of Sprague Dawley rat offspring whose mothers were administered DHEP orally during gestation [44]. Lara et al. (2017) suggested that histological abnormalities could result from rupture of the seminiferous cords, causing ectopic Sertoli cells and germ cells, and that more severe forms of dysgenesis may be due to exposure in critical phases of differentiation (masculinization programming window) [46]. More recently, Hu et al. (2018) reported that fetal testis Leydig cell development was disrupted by exposing pregnant Sprague Dawley rats to various doses of diethyl phthalate (DEP), DHEP or DEP + DHEP. Interestingly, fetal Leydig cells appeared to reduce in size and cytoplasm/nuclear ratio but significantly increase in number compared to controls; their tendency to distribute in large clusters confirmed the observations from previous studies, and the downregulation of enzyme genes related to steroidogenic action and the parallel decrease in INSL3 and CYP11A1 protein levels underline their dysfunctional status [47]. 

Exposure to phthalate esters has also been associated with altered Sertoli cell adhesion to gonocytes [45,48]. Other histological abnormalities, such as the presence of multinucleated cells and abnormal peritubular myoid cell distribution, might also be related to the action of phthalate esters, which seem to trigger abnormal gonocyte cell division and changes to the seminiferous tubule structure [45]. The most interesting aspect seems to be changes to the Leydig cells and their interstitial distribution in clusters. Hypothetically, phthalate esters could functionally change the ability of fetal Sertoli and Leydig cells to interact. As fetal Leydig cells are not under pituitary control, paracrine factors secreted by the Sertoli cells seem to control their differentiation and function, and Leydig cell clusters could thus result from disrupted Sertoli cell function induced by exposure to phthalates. Alternatively, these clusters and the proliferation of fetal Leydig cells with a reduced volume and cytoplasm/nucleus ratio could compensate for the disrupted steroidogenesis induced by phthalate esters [45]. Exposure to phthalate esters (DBP) in pregnant Wistar rats seems to cause not only fetal cell dysfunction and, hence, reduced fetal intratesticular testosterone levels but also a reduction in the proliferation and function of adult Leydig cell precursors (which normally only differentiate on puberty), with normal or reduced blood testosterone concentrations and increased LH [49]. In any case, all these changes could lead to impaired adult testicular function, hypospermatogenesis and infertility. 

Regarding BPA pre-natal effects, evidences are somewhat less represented than phthalates esters, but they seem related to alteration of steroidogenesis and INSL3 expression and, possibly, cryptorchidism. In particular, in vitro exposure of testicular cell cultures to BPA is associated with a reduced testosterone concentration and INSL3 mRNA [50]. A similar result was later observed in another study, confirming the dose-dependent nature of BPA interference with INSL3 and steroidogenesis. However, the authors noted that the possibility of a different response between humans and animal models should be taken into account [51]. A more recent study by Lv et al. (2019) further confirmed these results, finding a downregulation of the mRNA and protein levels of some steroidogenic enzymes and AMH transcribed and translated by Leydig cells and Sertoli cells, respectively. In addition to the reduced proliferation of fetal Leydig cells, the authors also hypothesized that exposure to these phenol compounds might be involved in the impaired development of the fetal testicle [52]. Key steroidogenic enzyme Cyp11a expression also seems reduced in a dose dependent way from prenatal BPA exposure [53].

There is also evidence of marked histological alterations of male reproductive tract in the offspring of Sprague Dawley rats exposed to various concentrations of BPA and analogues: in particular, the authors detected reduced seminiferous tubules diameters in post-natal male pups and increased oxidative stress markers [54].

In vivo, only an inverse correlation between INSL3 and BPA was found in umbilical cord blood from a cohort of neonates comprising 52 cryptorchid subjects and 128 non-cryptorchid controls; however, no correlation was revealed between testosterone and BPA [39]. Although the causality between reduced INSL3 and cryptorchidism could not be demonstrated and BPA levels during fetal life were not measured, the study stressed that the INSL3 pathway should be investigated when studying the in vivo effects of these environmental endocrine disruptors.

A recent study from Wei et al. confirmed the association between reduced testosterone and BPA administration to female pregnant rats: doses of 20 mg/Kg of BPA appeared able to induce in the male offspring reduction in testosterone and increase in estradiol levels, related to an increased estrogen receptor (α and β) expression. Interestingly, in the offspring BPA was also associated with a reduction of DNA methyltransferase transcription and increased pro-apoptotic activity (increased expression of caspase 7 and 9, bax; reduced expression of bcl-2). This allowed the authors to hypothesize that BPA could induce apoptosis [55]. This hypothesis is further strengthened by other observations of TUNEL positive cells in testis of the male offspring of BPA exposed animals [56,57,58]. 

It should be stressed that BPA induced alterations have been investigated with relatively high doses in animal models. A recent study from Dere et al. demonstrated that BPA reproductive toxicity on animal offspring is evident only at the highest doses (250,000 μg/kg/die) [59]. 

In conclusion, from the above evidence it seems clear that early in utero exposure carries a risk of urogenital developmental abnormalities, such as hypospadias, reduced anogenital distance, testicular gonadal histopathological alterations and various fetal testicular cell alterations. It is reasonable to suppose that these effects could co-exist and act in synergy in vivo, manifesting as a continuous spectrum of “exposure disorders” of which a future consequence is reduced semen quality.

### 2.2. Postnatal Exposure: Are There Repercussions for Adult Testicular Function? 

It should be stressed that there is currently no proof of the association between in vivo neonatal exposure to bisphenols and phthalates and impaired spermatogenesis and infertility in adulthood. The few published studies predominantly report results from in vitro studies or animal models. Atanassova et al. (2000) reported that a subgroup of mice exposed to high doses of phenols (including BPA) from 2 to 12 days post-partum did not show significantly altered reproductive behavior or infertility as adults, although from days 18 to 25 of life they showed an increase in the number of Sertoli cells and nuclear volume, elevation of the germ cell apoptotic index and greater pubertal spermatogenic activity [60]. A recent study by Meng et al. also investigated perinatal exposure to BPA in mice. Notably, during lactation, prolonged exposure to water contaminated with 0.2 or 2 μg/mL of BPA was not associated with any changes in epididymal or testicular volume compared with the control group. However, the male offspring of dams exposed to 2 μg/mL BPA showed reduced testosterone and sperm concentration and an increase in abnormal cells compared with controls [61]. Another interesting result from this study was the finding of a change in the inflammatory pathways in the groups exposed to the highest dose of BPA (increase in TNFα and higher NFkB and TLR4 protein expression). BPA appears able to elicit changes in cytokine secretion by binding TLR in adipocytes and by activating the JNK/STAT3/NF-κB inflammatory pathway [62]. There is also evidence of cytokine modification and ERK phosphorylation after Sertoli cell exposure to BPA [63]. All this suggests that induced inflammatory modifications might also impair testicular function and spermatogenesis. Similarly, Ogo and colleagues investigated exposure of male Wistar rats to BPA during the prepubertal period [64], finding an increased leukocyte infiltration on histopathological epididymal stains, an increased neutrophil recruitment (measured through myeloperoxidase assay) and increased epididymal IL6 concentration in the group exposed to the highest concentrations of BPA (200 µg/kg). The authors suggested that BPA is capable of hindering postnatal epididymal development as part of an induced inflammatory response. 

Inflammatory changes in the epididymis are likely to disrupt sperm maturation, potentially leading to infertility. Little information is available about the association between phthalates and inflammation. The few studies that do exist [65,66,67] do not suggest how it might affect reproductive development in male offspring. More recently, several authors reported that exposure of neonatal mice to BPA can disrupt pituitary development, with critical effects on their pubertal and hence gonadal development. The described effects comprise a measurable downregulation of the mRNA expression of several genes (including *pomc* and *Icam5)* involving different estrogen-related pathways, with sex-specific differences [68,69]. Furthermore, there is evidence from an animal study that the spectrum of reproductive alterations induced by chronic BPA exposure includes reproductive hormone alterations (increased gonadotropins and estradiol, reduced testosterone), spermatogenesis hindrance and increased oxidative stress [70].

A number of studies have exposed animals to mixtures of phthalate esters. One study investigated male Sprague Dawley rats exposed to low doses of a phthalate ester mixture (dimethyl phthalate (DMP), DEP, DBP, benzyl butyl phthalate (BBP), DEHP and di-n-octyl phthalate (DNOP)) (from 0 to 160 mg/kg/day) [71], finding a reduction in serum and testicular testosterone, reduced LH, an increase in histological seminiferous tubule abnormalities and downregulation of the main enzymes involved in testicular steroidogenesis in comparison with controls. All these changes were directly correlated with the total dose of phthalate esters. An earlier study reported similar changes in hormone levels, histological findings and steroidogenesis in male Sprague Dawley rats after exposure to DHEP alone, but at higher doses (from 250 to 750 mg/kg/day) [72]. This further underlines how these compounds, while having different chemical structures, can act through the same pathways and have an additive or synergic effect. It is plausible that simultaneous in vivo exposure to multiple compounds (even of different chemical species) may act together to disrupt testicular function.

Spermatogenesis may also be affected by causes other than the disruption of hormone action. In their above-cited study, Ha et al. also found an increase in cellular oxidative stress (reduced activity of superoxide dismutase and glutathione peroxidase and increased lipid peroxidation) [72]. Similarly, in a previous study pubertal Sprague Dawley rat testes cultured with mono(2-ethylhexyl) phthalate (MEHP) showed alterations in Sertoli cell adherence and tight junctions and increased oxidative stress, as evidenced by an increase in lipoperoxides and a decrease in glutathione levels [73]. Another study found that oral administration of various doses of DBP to adult male albino rats was associated not only with the hormone and semen changes already mentioned but also with a dose-dependent increase in seminal oxidative stress (reduced total antioxidant capacity) in comparison with healthy controls. Exposure to the highest doses (600 µg/kg) also provoked histological alterations to the seminiferous tubules, namely absence of spermatogenic cells in some of the tubules and tubular necrosis [74]. In a later study, adult male Wistar rats exposed to different concentrations of DBP via intraperitoneal injection presented dose-dependent seminal, steroidogenic and histological alterations with a reduction in the number of conceived embryos, an increase in abortions and a reduction in litter size compared with unexposed healthy controls [75]. 

Taken together, these observations could suggest that in the post-embryonic phase, phthalate esters and phenols (BPA) act on the already-differentiated genital structures, with their effects predominantly manifested through hormone interference. This deleterious interaction could take place both locally and centrally. Prepubertal exposure could be manifested through impaired maturation of the HPG axis and consequent disrupted maturation of the testicular (tubular and interstitial), epididymal and vesicular structures. Locally, some of the reported studies show that these EDCs can affect the seminiferous epithelium, inducing structural and functional alterations to the cell junctions and inflammatory damage resulting in tubular and epididymal histological changes that, at least in part, may be mediated by increased oxidative stress. Although evidence in humans is weak, it could therefore be hypothesized that these inflammatory alterations and increased oxidative stress in the critical period of pubertal development, combined with impaired function of the hormonal axis, could cause chronic changes to the genital tract. Given the transition from prepuberty to adulthood, the consequences may be less anatomically evident, but would probably have more repercussions on adult hormone function and/or spermatogenesis.

## 3. Clinical Data: The Real-Life Impact of BPA and Phthalates on Testicular Function 

Evidence of the impact of EDCs on post-pubertal spermatogenesis and testicular function is more contradictory, probably due to the difficulty of investigating them in vivo (Table 1 and Table 2). In 2016, Sathyanarayana and colleagues confirmed the correlation between exposure to EDCs and congenital malformations in vivo, demonstrating that the concentration of DHEP metabolites in urine samples from 371 women in the third trimester of pregnancy was associated with an increased incidence of genital abnormalities (hydrocele, cryptorchidism and hypospadias) in their male offspring [76], but the authors did not investigate potential testicular function and hence the future fertility of these boys. Hart et al. (2018) attempted to overcome this shortcoming by investigating testicular volume, sperm parameters and reproductive hormones in a cohort of 216 young men with known prenatal exposure to several phthalates’ esters. In particular, the authors found positive associations between maternal monoethyl phthalate (MEP) and adult semen volume, between maternal DEHP, DiNP (and their metabolites) and total testosterone, and a negative association between maternal mono(carboxyisooctyl) phthalate (MCiOP) and adult sperm motility [77]. However, results from this study need confirmation by further investigations, with a larger prospective caseload.

Exposure in adults generally seems to be compatible with impaired spermatogenesis and hormone function, but data on this association are limited, complicated by methodological issues and, in part, contradictory. In 2017, a meta-analysis found that the available data did not demonstrate a significant increase in male reproductive alterations following exposure to EDCs [94]. However, the evidence for rapidly metabolized EDCs such as BPA and phthalates was inadequate and most of the epidemiological studies included in the analysis found organochlorides or persistent organic pollutants (POPs) in various body fluids and a clinical correlation with reproductive diseases. Furthermore, the extreme heterogeneity of the studies and the large number of chemicals investigated individually mean that while their results could offer a general overview of the exposure profile, they did not enable the calculation of a reliable estimate of risk. 

In relation to testicular function, it is worth underlining the impact of EDCs on spermatogenesis. In 2012, Joensen et al. investigated 881 healthy young men (mean age 19.5 ± 1.3 years), finding through the use of regression models that their semen parameters were not significantly associated with the urine concentrations of various phthalate esters [78]. Similarly, associations between phthalate metabolites and sperm parameter were not found in a multicenter investigation of multiple environmental contaminants (phthalate esters, perfluoroalkyl acids, organochlorine compounds, heavy metals), although the authors found a negative association between PCB-153 and sperm motility [80]. Han et al., instead, found a weak association between urinary MBP and the presence of a pathological sperm concentration (OR 1.97; 95%CI 0.97–4.04) in a cohort of 232 men of unknown fertility status. The authors also investigated functional sperm parameters such as DNA integrity, but could not find any significant association with phthalate esters [79]. Axelsson et al, in a later investigation, did not confirm any association of sperm concentration or total sperm number to urinary phthalate esters concentrations; however, the authors detected a significant negative association between DEHP metabolites (MECPP in particular) and sperm motility [82].

In 2016, Wang et al. conducted a cross-sectional study of 687 infertile men, finding a negative correlation between phthalate concentration and seminal fluid volume, kinetic parameters (curvilinear velocity, straight line velocity) and normal forms, but no association with hormone parameters [81]. A subsequent study found an association between some phthalates and sperm DNA damage using a neutral Comet assay: the increase in sperm DNA fragmentation was proportional to the urine concentration of DHEP metabolites [83]. In contrast, a case-control study by Liu et al. (2017) on 139 infertile subjects vs. 150 fertile controls found higher urinary concentrations of various phthalate esters in the fertile group than in the infertile group [85]. 

Chen et al. (2017) investigated associations between 13 urinary phthalates and semen and hormone properties in a group of 796 Chinese students, finding various significant correlations between EDCs and the seminal and hormonal variables considered. Associations with altered semen parameters were found even in subjects with reported levels of daily exposure to phthalates below the United States Environmental Protection Agency’s reference limits. The authors also reported improved semen parameters (volume, normal forms) after a reduction in exposure due to the students’ relocation to another campus [86]. 

A multicenter American study investigated the urinary concentrations of various phthalate esters in 420 fertile men (aged 32 ± 6 years), finding an inverse correlation only between MBzP and sperm motility [84]. A single-center study conducted in 2018 on 599 men from couples about to undergo IVF/ICSI attending an assisted reproduction center found an inverse correlation between some phthalate esters and sperm concentration, but not with motility or abnormal forms [87]. 

In relation to the impact of EDCs on sex hormones, Joensen’s above-cited study found direct correlations between the main urinary metabolite of DHEP (MEHP) and total and free testosterone, not accompanied by any changes in gonadotropins [78]. These results were similar to those of a previous study by Meeker et al. (2008) [95], but were in contrast with Lenters et al (2015) who found a negative association between DiNP metabolites and total testosterone levels [80]. This evidence could indicate that phthalate esters may affect testicular function both directly, by disrupting steroidogenesis and testosterone synthesis, and centrally, by reducing the pituitary response of the hormonal axis. However, this aspect would merit further investigation, as gonadotropin levels in vivo could also be influenced by other factors (previous andrological disorders, treatments, other EDCs, etc.). Additional studies on human testicular cellular modifications induced by phthalate esters are also warranted, as the few available evidences do not demonstrate in vitro modifications of human steroidogenesis [96].

Possible BPA negative impact on sperm parameters has been investigated in vivo in several recent papers, although data is conflicting as there are papers indicating a negative correlation with sperm concentration and/or total sperm number [89,90], an association with alterations of sperm motility [88] and kinetics parameters [91], negative associations with sperm normal forms [92] or could not find any association with sperm parameters [93].

Indirect evidence of impaired endocrine function after exposure to BPA and analogs (Bisphenol S, Bisphenol E, Bisphenol F, Bisphenol B and BPA-diglycidyl ether) comes from a recent study by Desdoits-Lethimonier et al. in which cell cultures were prepared from testicles explanted from subjects with prostate cancer who were not under hormone treatment. The authors found a dose-dependent testosterone reduction in the culture medium after exposure to BPA and some analogs. Leydig cell production of INSL3 was increased after exposure to BPA, bisphenol B and S, and reduced after exposure to bisphenol E; both effects were dose-dependent. In contrast, Sertoli cell activity as measured by INHB levels was unchanged [97]. The more conflicting results than animal models can be in part explained by the known concept of species differences in BPA response among animal models can be broadened to humans as well, especially because human exposure in vivo differs greatly from the setting of controlled experiments, implying lower levels of overall BPA contaminations but arising from multiple exposure routes; furthermore, these papers investigated only urinary BPA, while it is now accepted that simultaneous contamination of mixtures if EDCs may mediate most of the reproductive alterations. In addition, these studies used different population and outcomes, increasing difficulty in making generalizations; in particular, difficulties arise when considering studies using caseloads of infertile subjects, as the supposed variability in sperm parameters may hide correlations with BPA levels. 

## 4. Conclusions

**Figure 1:**
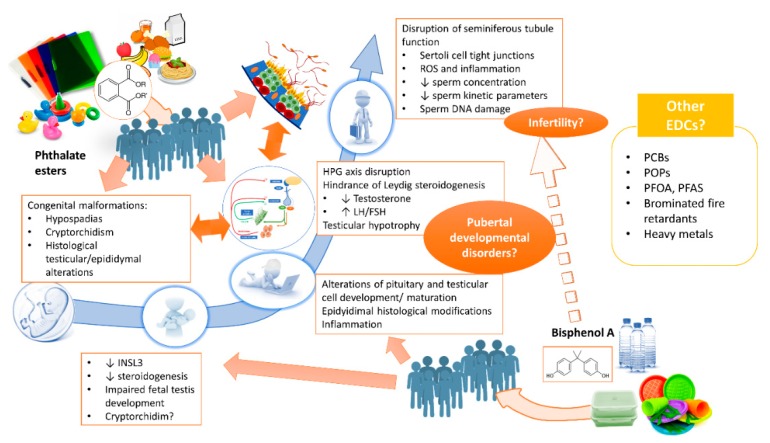
Effects of BPA and phthalates on the development and function of the male reproductive tract. Abbreviations: polychlorinated biphenyls (PCB), persistent organic pollutants (POP), polyfluoroalkyl acids (PFAS), perfluorooctanoic acid (PFOA).

The considerable scientific interest in EDCs in recent years has led to the collection of a large body of evidence on their mechanisms of action and potential risks for human health. In relation to reproductive health, many studies have focused on the effects of chemicals such as BPA and some phthalate esters whose exposure profile may be considered continuous and constant, given their ubiquitous distribution. For this reason, male fetuses are already exposed in intrauterine life, and data from animal models demonstrate that BPA and phthalates are associated with an increased risk of urogenital developmental abnormalities (hypospadias, cryptorchidism, histological alterations of the fetal testis). Postnatally, the main effects of these chemicals seem to occur through central and peripheral disruption of hormonal homeostasis. These hormonal changes could inhibit the proper development of the male genital tract, inducing chronic modifications, in part mediated by inflammatory mechanisms and oxidative stress. Epidemiological data on in vivo exposure to BPA and phthalates are more difficult to interpret, although they are generally compatible with the congenital malformations and impaired spermatogenesis and hormone function found in vitro (Figure 1).

It can be hypothesized that in vivo, BPA and phthalates act in synergy and that their effects are manifested as a continuous spectrum of “exposure disorders”, further modulated by other factors such as the period of exposure (prenatal, prepubertal or adulthood) and any concomitant exposure to other EDCs, and that a consequence could be impaired testicular function (hypogonadism, reduced semen quality). However, the logical consequence of an increased risk of infertility is still difficult to demonstrate in vivo.

## Figures and Tables

**Table 1:** Summary of recent available clinical evidence of phthalate esters effects on sperm parameters and reproductive hormones.

Paper	Year	*N* subjects	EDCs investigated	Subjects with EDCs >LOD	Population	Sperm Parameters	Hormones
Joensen et al. [78]	2012	881	urinary MEP, MnBP, MiBP, MBzP, MEHP, MEHHP, MEOHP, MECPP, MOP, MCPP, MiNP, MHiNP, MOiNP, MCiOP	MOP 2.5%; others 79.6–100%	general population	semen parameters were not significantly associated with the urine concentrations of various phthalate esters	urinary MEHP was associated with total and free testosterone, but not with gonadotropins
Han et al. [79]	2014	232	MBP, MEP, MEHP, MBzP, phthalic acid	MBzP 8.9%; MEHP 58.9%; MEP 77.6%; others 100%	men with unknown fertility attending a fertility center	weak association between MBP and sperm concentration (OR 1.97; 95%CI 0.97–4.04); no associations with sperm DNA integrity	no correlations between phthalates and serum hormones after adjustments for confounders
Lenters et al. [80]	2014	602	DEHP, MEHHP, 5OH-MEHP, MEOHP, 5oxo-MEHP, MECPP, 5cx-MEPP, DiNP, MHiNP, 7OH-MMeOP, MOiNP, 7oxo-MMeOP, MOiCP, PFAS, PFOS, PFOA, PFHxS, PFNA, PFDA, PFUnDA, PFDoDA, cadmium, lead, mercury, PCB153, *p*,*p’*-DDE, HCB	phtalates 39–100%; metals 100%; perfluoroalkylic acids 29–100%; organochlorines 93–100%;	male partners of pregnant women	no associations between phthalates and sperm parameters; PCB-153 negatively associated with sperm motility	DiNP metabolites negatively associated with testosterone
Wang et al. [81]	2015	687	semen plasma MMP, MEP, MBP, MBzP, MEHP, MEHHP, MEOHP and MOP	MOP 13%; MBzP 29%; MMP 35%; others 67–100%	men with unknown fertility, attending a fertility clinic	negative associations with semen volume semen volume (MBP, MEHP, MEHHP, MEOHP), sperm kinetics parameters (MBzP, MEHP), normal forms (MBzP)	no associations with reproductive hormones
Axelsson et al. [82]	2015	314	urinary and serum MEHP, MECPP, MEHHP, MEOHP, MCiOP, MHiNP, MOiNP, MBP, MBzP, MEP	>97%	general population	DHEP metabolites and MECPP in particular were negatively associated with sperm motility; MEHP was positively associated with HDS.	N/A
Wang et al. [83]	2016	1040 (483 reproductive hormones; 509 DNA integrity; 467 sperm apoptosis)	MMP, MEP, MBP, MBzP, MEHP, MEHHP, MEOHP, MEHP urinari	>90%	men with unknown fertility, attending a fertility clinic	MEHP, MEHHP, MEOHP: association with increased DNA fragmentation and apoptosis	MEHP: negative association with Estradiol, total and free Testosterone.
Thurston et al. [84]	2016	420	urinary MEHP, MEHHP, MEOHP, MECPP, MBP, MiBP, MCPP, MBzP, MEP	77–100%	partners of pregnant women	MiBP: positive association with motility; MBzP: positive association with total sperm count	N/A
Liu et al. [85]	2017	139 infertile vs. 150 fertile	urinary MMP, MEP, MBP, MBzP, MEHP, MEHHP, MEOHP	MMP 3.5%; MBzP 50.9%; others >99%	infertile vs. fertile subjects	MEHHP was significantly riduced in cases vs. fertile controls	N/A
Chen et al. [86]	2017	796	urinary MMP, MEP, MiBP, MnBP, MCHP, MCPP, MnOP, MEHHP, MECPP, MEOHP, MEHP, MBzP, MiNP	MCHP 2.5%; MiNP 17.3%; MnOP 32.5%; MBzP 35.6%; others >88%	general population	negative associations with sperm volume (MiBP, MEHP), concentration (MEP), motility (MEP, MnBP, MCPP, MnOP), normal forms (MEHP, MnOP, MBzP)	negative associations with estradiol (MnOP, MEHHP, MECPP, MEOHP), testosterone (MMP, MiBP, MnBP, MEOHP); positive associations with gonadotropins (MBzP)
Al-Saleh et al. [87]	2019	599	MEP, MiBP, MnBP, MBzP, MECPP, MEHHP, MEOHP, MEHP	MBzP: 26.1%; others: >96%	infertile	MECPP, MEHHP, MEOHP and calculated ΣDEHP: positive association with sperm concentration; calculated excreted %MEHP: negative association with sperm concentration	MiBP and MEHHP were inversely associated with testosterone and FSH, respectively. Estradiol was positively associated with MEP. %MEHP was positively associated with gonadotropins (FSH, LH)

Abbreviations: monoethyl phthalate (MEP), mono-n-butyl phthalate (MnBP), monoisobutyl phthalate (MiBP), monobenzyl phthalate (MBzP), mono(2-ethylhexyl) phthalate (MEHP), mono(2-ethyl-5-hydroxyhexyl) phthalate (MEHHP), mono(2-ethyl-5-oxohexyl) phthalate (MEOHP), mono-(2-ethyl-5-carboxypentyl) phthalate (MECCP), mono-n-octyl phthalate (MOP), mono-(3-carboxypropyl) phthalate (MCPP), monoisononyl phthalate (MINP), mono(hydroxyisononyl) phthalate (MHiNP), mono(oxoisononyl) phthalate (MOiNP), mono(carboxyisooctyl) phthalate (MCiOP), mono-(2-ethyl-5-carboxypentyl) phthalate (5cx-MEPP), mono-(4-methyl-7-hydroxy-octyl)phthalate (7OH-MMeOP), mono(4-methyl-7-carboxyheptyl) phthalate (MOiCP), polyfluoroalkyl acids (PFAS), perfluorooctane sulfonic acid (PFOS), perfluorooctanoic acid (PFOA), perfluorhexane sulfonic acid (PFHxS), perfluorononanoic acid (PFNA), perfluorodecanoic acid (PFDA), perfluoroundecanoic acid (PFUnDA) and perfluorododecanoic acid (PFDoDA), polychlorinated biphenyls (PCB), dichlorodiphenyldichloroethylene (DDE), hexachlorobenzene (HCB).

**Table 2:** Summary of recent available clinical evidence of Bisphenol A effects on sperm parameters and reproductive hormones.

Paper	Year	*N* subjects	EDCs investigated	Subjects with EDCs >LOD	Population	Sperm Parameters	Hormones
Lassen et al. [88]	2014	308	urinary BPA	98%	general population	highest BPA concentration quartile had reduced % progressive motility vs. lower quartile.	highest BPA quartiles had higher LH, testosterone and estradiol compared to lower quartile.
Knez et al. [89]	2014	149	urinary BPA	98%	infertile	BPA: negative association with sperm concentration and vitality	N/A
Adoamnei et al. [90]	2018	215	urinary BPA	95%	healthy volounteers	negative association between BPA and sperm concentration, total sperm count	positive association between BPA and LH
Ji et al. [91]	2018	500	urinary BPA	73.6%	fertile	BPA was associated with several CASA kinetics parameters (positive associations: LIN, STR, WOB; negative associations: ALH))	N/A
Pollard et al. [92]	2019	161	urinary BPA	87%	men with unknown fertility	abnormal morphology was associated with higher BPA mean urinary concentrations	N/A
Kim et al. [93]	2019	146	Urinary, semen plasma, blood BPA	43.1% (urine); 71.3% (semen plasma); 77.6% (blood)	infertile	no associations with sperm concentration and motility; no significant effect on embryo embryo quality, oocytes retrieved, pregnancy	N/A
